# Feeling anxious’- women’s experiences of having a baby in Australia during the COVID-19 pandemic using the Voqual real time app

**DOI:** 10.1186/s12884-023-05993-9

**Published:** 2023-09-19

**Authors:** Hazel Keedle, Kimberley Tomczak, Belinda Lequertier, Hannah G Dahlen

**Affiliations:** 1 School of Nursing and Midwifery, Western Sydney University, Locked Bag 1797, Penrith, NSW 2751 Australia; 2 Molly Wardaguga Research Centre, School of Nursing and Midwifery, Charles Darwin University, Level 11, 410 Ann Street, Brisbane, QLD 4000 Australia

**Keywords:** Pregnancy, Maternity models of care, Anxiety, COVID-19, Antenatal, Midwifery

## Abstract

**Purpose:**

Internationally, the COVID-19 pandemic impacted maternity services. In Australia, this included changes to antenatal appointments and the reduction of support people during labour and birth. For women pregnant during the pandemic there were increased stressors of infection in the community and in hospitals along with increased periods of isolation from friends and families during lockdown periods. The aim of this study was to explore the real-time experiences of women who were pregnant and had a baby during the first wave of the COVID-19 pandemic in Australia.

**Methods:**

This study followed seven women throughout their pregnancy and early parenthood. Women created audio or video recordings in real time using the Voqual app and were followed up by in-depth interviews after they gave birth.

**Results:**

Using narrative analysis their individual stories were compared and an overarching theme of ‘feeling anxious’ was found which was underpinned by the two themes ‘model of care’ and ‘environment’.

**Conclusions:**

These findings highlight the protective impact midwifery continuity of care has on reducing anxiety in women during the pandemic, and that the home environment can either be secure and safe or a place of isolation.

## Background

Women having a baby during the COVID-19 pandemic shared many of the experiences and concerns of the broader adult population, including the threat of infection and the impact of public health measures, such as increased feelings of isolation due to lockdowns [1, 2]. However, childbearing women faced additional pandemic-related stressors, such as difficulty accessing appropriate information on COVID-19, and concerns about the safety of attending antenatal appointments and birthing in the hospital environment [3, 4]. At the same time, health service measures to reduce the risk of COVID-19 infection has resulted in fewer face-to-face appointments, and restrictions on partners and support people attending antenatal appointments, ultrasounds or the birth [4–6]. Consequently, giving birth during COVID-19 has been characterised by uncertainty, loneliness and distress [5], and women have reported feeling abandoned and alone due to maternity care changes [7].

Previous studies have investigated changes to maternity care during the pandemic in Australia through surveys with women [5, 7] and a survey and interviews with midwives [6]. At present, there is a lack of literature regarding pregnant women’s experiences throughout the COVID-19 pandemic using real-time data collection methods. A smart phone app called “Voqual” was developed for real-time audio or visual data collection and instantaneous researcher access to the data without the need for multiple follow up interviews or risking memory recall biases [8, 9]. Moreover, chronic stress, such as that experienced over the course of the COVID-19 pandemic, has been shown to impair memory retrieval, owing to high cortisol levels [10].

The use of the Voqual app in this study provided a unique insight into how women experienced maternity care throughout the first wave of the COVID-10 pandemic. As such, the aim of this study was to explore the real-time experiences of women who were pregnant and had a baby during the first wave of the COVID-19 pandemic in Australia.

## Methods

The Birth in the Time of COVID (BITTOC) study was a mixed-method, longitudinal study investigating the pandemic-related experiences and mental health of 3,191 women who were pregnant and gave birth during COVID-19 in Australia from March 2020-to February 2021, and the impact of these experiences on the development of their children (2, 6, 12, 24 months following the birth) [11] Mixed methods research has the capability to give greater understanding through the integration of a variety of qualitative and quantitative methodologies and analysis [12, 13].

Women were invited to be interviewed through social media advertising between April and May 2020. Women who were currently pregnant were also invited to participate in the Voqual real time section of the study. The Voqual real-time app, a smartphone application, enabled participants to provide regular audio or video recordings, journaling their experiences in real time, capturing the immediate thoughts and experiences of participants [8]. Prior to recruitment, ethics approval was sought and obtained through Western Sydney University Human Ethics Committee, ethics number H13825.

### Feminist framework

A feminist theoretical framework informed the study design. Using the Voqual app, participants decided whether they would make a recording, when they would make a recording and what information they would include. For example, the app allows participants to remove recordings if they later decide they do not want them included in the data, placing the power and control with the participant and not the researcher [14, 15].

An important aspect of qualitative feminist research is the role of reflexivity and identifying the roles and subjectivity of the researchers [16, 17]. The recruitment and interviews were undertaken by HK, a midwifery researcher, who mentored KT, an undergraduate Bachelor of Midwifery student undertaking a summer scholarship research project. Analysis was undertaken by HK and KT with regular reviews with HD, the chief investigator of BITTOC.

### Participants

Women interested in joining the Voqual study were given a participation information sheet and consent form and instructions for accessing and using the Voqual app. Participants were asked to make regular recordings during their pregnancy and share their experiences of pregnancy and maternity care during the pandemic. The guidance on what topics to record was purposively kept broad to encourage the woman to share what she felt was important at the time. A total of seven participants made 43 recordings from around twenty weeks of pregnancy to six months postnatally (Table 1).

**Table 1:** Recordings and interviews

Pseudonym	Number of Recordings	Range of length of antenatal recordings (minutes)	Postnatal interview length (minutes)
Amelia	1	9:42	42:57
Emma	2	1:01–2:01	45.54
Charlotte	2	2:00–4:11	49:22
Ava	3	0:43–20:22	37.14
Mia	7	1:58 − 4:59	1 h 18 min
Sophia	12	1:13–4:41	55:21
Olivia	16	0:57 − 8:15	1 h 24 min

### Data collection

Participants provided a video or audio recording via the app which they chose to save and upload. The researcher accessed the recordings via the secure database and downloaded them as MP4 or MP3 files which were then stored on password protected cloud-based storage. The recordings were transcribed and deidentified for analysis. All women were interviewed post birth via zoom (age range 2 months to 8 months post birth). The researchers listened to all antenatal recordings from Voqual logs prior to individual interviews to develop individualised questions for each woman, additional to planned key questions (Table 2). For example, one woman made a comment in her antenatal recordings about being unable to shop for maternity clothes in physical stores due to lockdown and this was explored further during the interview.

**Table 2:** Interview questions

Questions for interviews
Can you tell me about your experiences of being pregnant/giving birth/early parenting during the COVID-19 pandemic?
What changes to your pregnancy care did you experience as a result of COVID-19?
How did COVID-19 restrictions impact upon your partner, family and/or social support?
What changes to your birth care did you experience as a result of COVID-19?
Did your maternity care differ from what you expected it would be?
What changes to your postnatal care and early motherhood did you experience as a result of COVID-19?
Did you make any active decisions yourself to change your pregnancy/birth/postnatal plans as a result of the COVID-19 restrictions?
What could your care provider/service have done to make your experience better?
COVID has had a huge impact – what might be some of the positive aspects of this pandemic in terms of provision of maternity care?

### Narrative analysis

Qualitative research explores participants’ experiences, behaviours and feelings through the sharing of stories [18]. Narrative analysis was chosen as the methodology as the focus was on individual stories that create meaning and understanding [19–21]. The first author had used narrative analysis when using the Voqual app in a previous study and found this methodology kept the stories of the individuals as whole rather than separating into parts as can be found in other qualitative analysis methodologies [22]. The transcriptions from the Voqual recordings and the individual interviews were collated for each participant, creating a chronological story across sequential timelines for analysis and exploration [23, 24]. The individual stories of participants were uploaded to the qualitative software NVIVO 12, where they were read, re-read and analysed. A synopsis was written on each story to summarise and identify the influences framing the narrative [25], and stories were compared for similar and contrasting themes. Regular team meetings occurred throughout the research process to discuss the analysis and findings.

## Findings

### Demographics of women

The participants were aged 30 to 39 years, university educated (undergraduate or postgraduate level) and in a long-term relationship (Table 3).

**Table 3:** Demographics

Pseudonym	Age range	Education level	Country of birth	State / Territory	Previous pregnancies / births	Baby’s age at interview
Amelia	35–39	Postgrad	Australia	City WA	1 / 1	8 months
Emma	Unavailable Data					6 months
Charlotte	30–34	Undergrad	Australia	Regional NSW	2 / 1	2 months
Ava	30–34	Postgrad	Australia	Regional NSW	4 / 2	1 month
Mia	30–34	Undergrad	Australia	City NSW	1 / 0	4 months
Sophia	30–34	Postgrad	Australia	City NSW	1 / 0	5 months
Olivia	35–39	Postgrad	UK	Regional NSW	1 / 0	2 months

## Themes

**Fig. 1:**
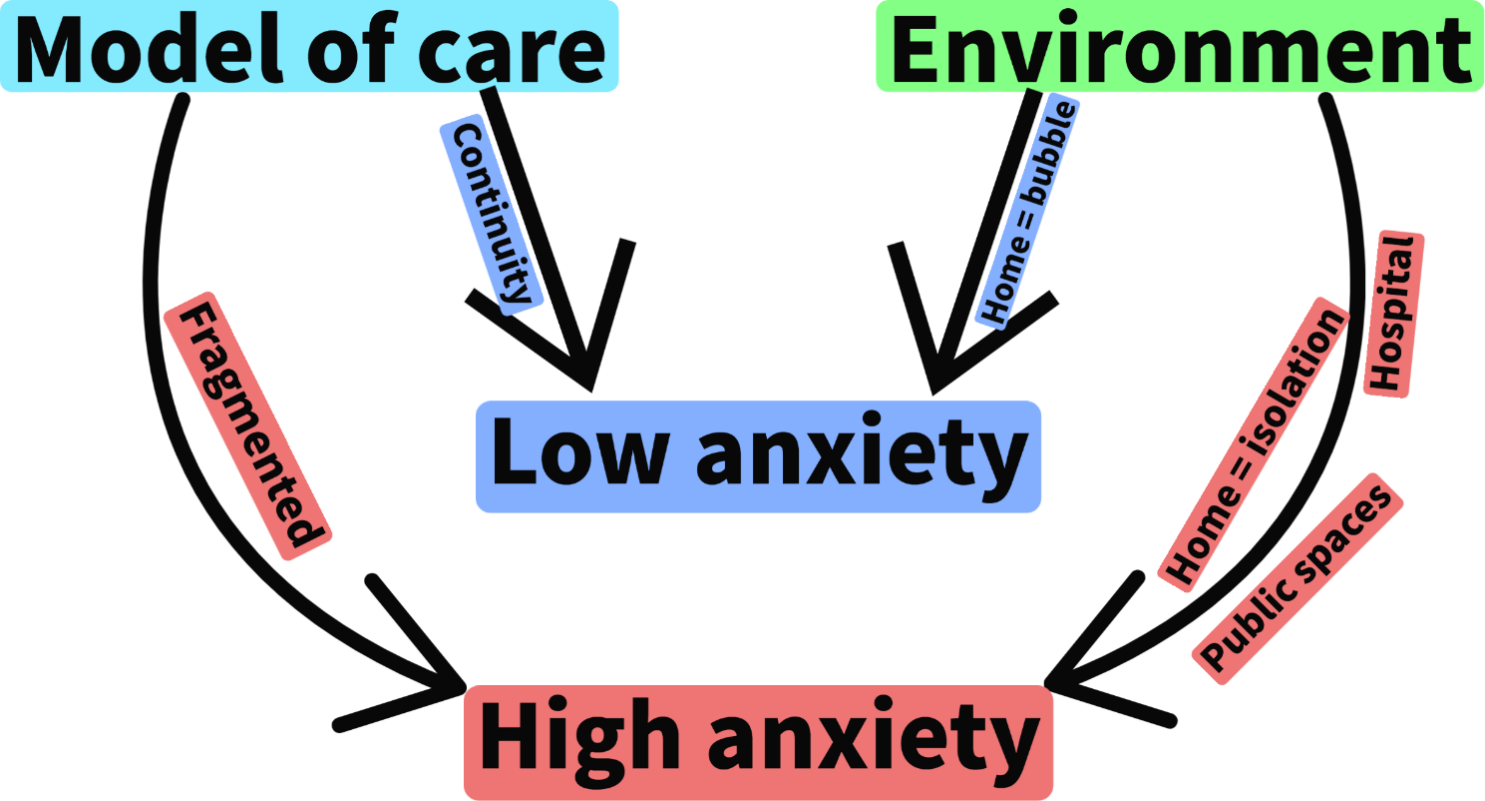
Concept diagram of themes

An overarching theme of ‘feeling anxious’ was identified, and this was informed by two themes: ‘model of care’ and ‘environment’ (Fig. 1).

Women experienced different levels of anxiety during the first wave of the COVID-19 pandemic and this impacted on how women experienced their pregnancy, birth and postnatal period. Issues such as lockdown measures, infection rates, pandemic news coverage and changes to antenatal appointments influenced anxiety levels and women shared this through their audio recordings. The feelings of anxiety were exacerbated or mitigated by the ‘model of care’ and ‘environment’.

The women in this study experienced a variety of **models of care, **which impacted how supported or anxious they felt during their pregnancy. Sophia, who had standard fragmented maternity care, experienced significant gaps in her medical care during pregnancy despite being identified as high-risk quite early in her pregnancy. In contrast Olivia felt safe and supported under the care of a privately practising midwife (PPM). When Olivia did access the public hospital, she described a chaotic and disorganised system, further highlighting to her the benefit of having a PPM.

Lockdown measures brought about big changes for women’s everyday **environment**. Women who worked outside the home were restricted to home or working in an environment under restrictions. Some women found the experience beneficial, and home became a sanctuary, and for others home became a prison of loneliness. Mia felt lonely throughout her pregnancy and postnatal period due to her partner working outside of the home as an essential worker. Emma however was able to continue to work as a midwifery student outside of the home, finding the increased personal protective equipment and screening added to her feelings of safety. Charlotte was able to plan for, and undertake, a homebirth, surrounded by her care providers and family, followed by six weeks postnatal care and time at home bonding with her newborn baby together with her husband and first child, creating a warm space, shut off from the anxieties of the COVID-19 pandemic. The four stories of Sophia, Olivia, Emma and Mia will now be explored further. Similar themes were seen across all seven women’s stories.

### Sophia

Sophia’s story details her perinatal experiences with the public hospital system during the pandemic and highlights a system in crisis allowing women to fall through cracks. Sophia’s **anxiety** increased due to experiencing a fragmented **model of care**. Sophia was pregnant throughout the first wave of COVID-19 in Australia and provides insight into how women were affected by changes that came in overnight, with antenatal appointments reduced and replaced with telehealth and restrictions placed on support people attending appointments. For Sophia, the move to telehealth for antenatal appointments resulted in missed tests and a delay in receiving her 20-week anomaly ultrasound results until 28 weeks. This caused Sophia significant **anxiety** as she had been identified as having a high-risk pregnancy and was unsure if she would be able to birth at her chosen hospital.

*“The woman on the phone tried to tell me that I went in for a 24-week check-up. I said “No, I didn’t have a 24-week check-up, all of my check-ups were cancelled.” She goes “oh but I have notes here and I’m well they are not my notes because I wasn’t there;” so that was really frustrating, they were trying to tell me that I had been in, or I had had extra phone consults where I know for a fact that I hadn’t” [Sophia, recording 6]*.

Sophia relied on calling a pregnancy advice phone number to supplement the lack of professional advice she was receiving and calm her **anxiety**.

Sophia suffered with constant **anxiety** about how she would cope, as she’d had a history of significant mental health concerns and admitted to feeling vulnerable in the hospital **environment**.

*“I’m still not allowed to have more than one person and it just makes me so mad, and you know I have a lot of mates who I see on Facebook who are all, it’s like- you know, I get to go back to the gym and I’m like how awesome for you. How great that you get to go back to the gym, and I have to give birth alone without my support network around me when really that should- that should just be a fricken fundamental right” [Sophia, recording 2]*.

Sophia was able to have her partner as her support person but during labour she experienced complications with bleeding and fetal heart rate abnormalities, and she was taken for an emergency caesarean. Sophia found that a difficult experience and very far from the birthing experience she was hoping for. During the operation Sophia struggled to have skin-to-skin with her baby resulting in Sophia feeling powerless and separated from her baby.

*“The hardest thing for me and I still- I still get choked up talking about it is when they brought him out I couldn’t even hold my head up to look at him, because I was so numb….And so, they whisked him away with my partner, oh that’s the other thing, I was like, I really wanted skin to skin and so as soon as he was out, Lachlan whipped off his shirt, did skin to skin with him, but then umm, when he finally came back over to me, when the midwife brought him over to me, after he was like all checked and everything umm, and she just held- held him cheek to cheek for like 20 minutes. Just held him at my face and but- again it was that thing of like, I couldn’t hold him, I- I had no feeling in my hands and arms, and it’s sort of like wow my first experience as a mother is just powerless, helpless, not able to do anything that is just so intuitive, and it was so hard.” [Sophia, Interview]*.

Sophia was interviewed when her baby was 5 months old. On reflection, Sophia highlighted some positives of having a baby in lockdown as her partner took some leave before returning to working in their home **environment** which gave Sophia extra support in caring for the baby.

*“he will take the baby in the morning, I wake up every single morning to an iced caramel latte on my bedside table, and then, you know when he starts work he will just sort of come in and put the baby next to me and at that- that really works for us, really works for us” [Sophia, Interview]*.

Following the birth there was a gradual lifting of lockdown restrictions, yet Sophia felt **anxious** that she would be seen as a bad mother if she took her baby outside of the home **environment**.

*“I’d be oh I really want to go out, you know, just go for a coffee but I’m like, oh my god with the pandemic am I doing the right thing, are people gunna be looking at me going ‘oh my god, you take your newborn out what’s wrong with you’, and so there was that whole new level of isolation, I found that now I had like this little screaming gremlin, and I just was like, I just want to go out for a cup of coffee but I don’t want people to think that I’m being, like nonchalant with his health or wellbeing” [Sophia, Interview]*.

Sophia was offered online parenting groups through community health but due to them being online she opted to build her community through her family and friends and attended a mums and bubs exercise group with her sister. Overall, Sophia regards her experience as being one of high stress and increased feelings of **anxiety**.

*“I wouldn’t wish pregnancy in a pandemic upon anybody. Like it has honestly made me never want to be pregnant again … there is no way I would want to put myself through that and be pregnant during a pandemic ever, ever again. I would not recommend - one star. One out of ten” [Sophia, Interview]*.

### Olivia

For Olivia, the COVID-19 pandemic impacted her initial decision-making regarding **model of care**. Early on in her pregnancy, Olivia had chosen to transfer her care from fragmented public hospital care to a PPM **model of care**. Significant concerns about lack of choice and the hospital **environment** impacted Olivia’s **feelings of anxiety** and prompted her decision to transfer to a PPM **model of care** and to birth in the home **environment**.

*“Going to the hospital during Covid hasn’t been great it’s been a bit chaotic and disorganised, there are temperature checks on the way in, all that kind of precaution stuff, then you get in the waiting room and, [laugh] and then you sit there for an hour with loads of people in close proximity, so it’s just really weird and bizarre. Umm, lots of things have been denied and been difficult to access, I’ve had to struggle to get a transvaginal scan to check out my cervix, because I have had two prior LEEP [excision on cervix due to cervical cancer] procedures, all of it’s a big faph [palava] and its left me with very little faith in the system down there. So, onwards towards a homebirth” [Olivia, recording 2]*.

Later recordings from Olivia focussed on being forced to attend a hospital booking in appointment to ensure the local hospital had her information in case she needed to transfer from home to hospital. The thought of attending this appointment increased her **anxiety** leading Olivia to cancel her appointment:

*“I’ve had to cancel my appointment by lying instead of just being completely open and honest about, I’m not coming because I know that I can book in, over the phone and I’ve got a privately practice midwife and I’m having a homebirth I do not need to come to the appointment. . I get why they want me to go in, in person for that but I have a feeling that I’ll go in there and I’ll answer those questions and I’ll be talking about the homebirth, and they just won’t listen and take my perspective and decision to have a homebirth as valid” [Olivia, recording 9]*.

By recording 10, Olivia was successful in completely transferring her care to a PPM which reduced Olivia’s **feelings of anxiety**.

*“I’m with my private practice midwife, which is good, and I feel a lot more calmer since I refused the intra-vaginal scan, which was unnecessary. . I want to be left alone kind of thing and Elizabeth [midwife] is coming on Monday actually so it’s just like a really good touch point that since transferring my care and having less monitoring in the form of scans, I feel a lot more confident and comfortable in being pregnant” [Olivia, recording 10]*.

Although Olivia mentions having to switch off social media and news that discussed the COVID-19 pandemic, due to increasing **anxiety** caused by so much COVID information, in later recordings, like Sophia she is less focussed on the evolving pandemic and more focused on preparing her home-birthing **environment**. For Olivia the private midwifery **model of care** resolved many of her concerns.

Olivia made her last recording the day after her baby was born where she described her experience of birthing at home.

*“I birthed my baby on the- in the bedroom hanging off, clinging off the bed on all fours in like 25 minutes of just pushing and I felt really in control and really empowered, and like I just made a conscious decision at some point when I was in the bath to make noise and grunt- grunt- grunt my way through labour. So, I didn’t have any tears and my partner caught the baby and, I birthed the placenta normally on the bed afterwards and everything was just really amazing and I feel good today” [Olivia, recording 16]*.

Through Olivia’s decision to have a homebirth she was able to connect to the local homebirth group that had a social media group page and met regularly. Although Olivia was on maternity leave, she was looking forward to returning to work.

*“I’ve got like, been able to have a really positive experience, Covid has helped with that decision making, and being able to be at home for work, I mean I could have probably stayed at home a lot anyway, cause I am sort of quite autonomous in my job. … So, like, its- it’s always been quite digital, I think in – in many ways like, Covid might help with, oh I dunno- cause like, going back to work, I might be able to work at home, more legitimately. But that blurred boundary of being able to work and care for a baby at the same time is gunna be quite difficult to navigate but at least I have got some greater flexibility I think, to be able to be at home more” [Olivia, interview]*.

Olivia’s **model of care** during pregnancy gave her security and a relationship with a midwife she relied on for information and support, which contributed to alleviating her **anxieties** about attending the hospital **environment** during a pandemic. Olivia was at risk of isolation and loneliness due to immediate family living overseas and the change to working from home, but through choosing homebirth Olivia gained access to a strong community of women both in her local area and online, which provided ongoing support following the birth of her baby.

### Emma

Emma’s experience of giving birth during the COVID-19 pandemic is one of relative calm due to negotiating her **model of care** and controlling her **environment**. Emma was a final year midwifery student during her pregnancy with her third child and had chosen to birth in the hospital she was training in under a fragmented **model of care**. However, Emma was able to negotiate having antenatal check-ups with a friend who was a registered midwife during Emma’s placements which provided a modified midwifery continuity **model of care**. Emma felt this was very personalised care and felt relieved she was still able to hear her baby’s heartbeat on a regular basis. If this option had not been available, Emma would have **felt anxious** regarding the wellbeing her baby.

*“I know a lot of the women that I was looking after had only had telehealth for most of their pregnancy by the end. And umm, yeah, I guess I would have felt concerned if I wasn’t getting the regular blood pressure checks or, you know that sort of- … I mean even just things like them checking the foetal heart every appointment, umm that was reassuring to me whereas if you weren’t coming in and getting a check like that you wouldn’t have felt that” [Emma, Interview]*.

Emma felt largely unaffected by the pandemic and was not stressed working in the clinical environment. However, public spaces raised her **anxiety** levels, so she avoided them. Emma described she was still able to undertake her midwifery placements and feeling highly protected by the patient and visitor COVID-19 screening procedures taking place before entry to the hospital.

Emma describes this as her “best birth” and felt supported by her modified midwifery led **model of care** in the public hospital she had placements in. This was in comparison to the private obstetric **model of care** she had experienced for her previous two births. For Emma her biggest concerns around the birth were the social restrictions having on her children meeting the new baby.

*“I mean the hardest- hardest thing was who was allowed to be in the hospital with you. Umm, when I had Evie it was still only one support person. Which was fine I only ever really want my husband there anyway, but it was after that, when I couldn’t have the kids come and because Evie was a big baby, I had to stay for blood sugar monitoring for 24 hours so, I already had been away from them for a night and then it was another night, so it was just- it was really hard, and I remember- really loving having my daughter come and meet my little boy when he was born, and that first meeting whereas we had to hold off, and wait until I got home, which I know affected a lot of people, like it was probably one of the hardest things” [Emma, Interview]*.

During her postpartum, Emma reported that the enforced social restrictions during the COVID-19 pandemic did not affect her physical or mental health as she was able to keep a family bubble within her home **environment**, enabling bonding without the interference of visitors.

*“I’m a bit of an introvert so I kind of liked having to [laugh] stay home. But umm, I just- I avoided public areas as much as possible, like I kept my kids home from school as well for- my daughter was already home-schooling, but my son’s kindy was still running but we kept him home. We just avoided shops, I just did a lot of like sending my husband out to get stuff, if we could online shop, I would do that. We just really would avoid, being around people, and if they were sick, we were absolutely nowhere near them. We just kind of kept to ourselves for a good few months. . with all of our kids kept into our little bubble…so, for us it wasn’t really too much different but it, we kind of just kept a lot more isolated” [Emma, Interview]*.

### Mia

Mia initially requested to be accepted into the local MGP program at the hospital but the program was full so she chose a shared **model of care** with her GP so she could spend less time at the hospital and build an effective therapeutic relationship with her care provider. However, the quality of the relationship with the health professional was unsupportive, leaving Mia feeling more **anxious** throughout the perinatal period. Mia’s first antenatal appointment was scheduled within days of a wave of COVID-19 infections and her anxiety was increased due to distrusting relationship with her GP.

*“My doctor, I didn’t really like her. She was, I wouldn’t go with her again, she was really curt, and again in those situations I didn’t really, yeah. The- they are not COVID related but I didn’t really feel like I was getting much from her or that I would ask- could ask a lot of questions … I did have another doctor before that, and then I moved to her because she was closer to me, I could walk to her, thing. I wouldn’t recommend her” [Mia, Interview]*.

The impact of lockdowns and rules around isolation resulted in changes in her local **environment** and these created feelings of **anxiety** for Mia, as they limited her choices around her daily activities, such as grocery shopping, especially as her due date approached. At 36 weeks Mia made the following comment about her concerns.

*“One of the things I’m bit worried about is having accidentally visited a place that turns into a hotspot and having to self-isolate, for me that’s not so much of a problem. But if my husband goes somewhere and he needs to self-isolate he will not be able to come to the birth. So, at the moment we are not going out and about we are avoiding you know going out to a restaurant or a café or anything like that and I’m even getting a bit worried about doing our groceries, so I think I’m going to get my mum to do our groceries this week” (Mia, recording 7)*.

During pregnancy Mia controlled her **environment** through a self-imposed isolation due to **feeling anxious** about being exposed to Covid-19. Mia’s family went grocery shopping for her and left it outside the door. Mia describes feeling ‘trapped’ inside her home for months on end without anything to do, as all her work had also been cancelled.

*“It’s a bit crazy to kind of voluntarily self-isolate, but we can still- we will still go out for walk, I mean I’m still happy to see small groups of family and friends face to face that’s fine. But yeah definitely anywhere that is going to require isolation, anywhere there is strangers and things like that will be a no go. For however long this baby takes to come out, so you know maybe a month maybe six weeks, maybe a week, who knows” (Mia, recording 7)*.

Working from home during pregnancy meant a change to Mia’s work **environment** which meant Mia missed out on socialising with colleagues and connecting with other pregnant women at work. This led to loneliness, exacerbating her **feelings of anxiety**.

*“So, I think probably the biggest thing for me at that point in time was like, loneliness and not being able to connect with other people really, because if I had have known these other people were pregnant at work as well, you know, we’d be talking about it all, and you know, catching up with each other and all that kind of stuff…Because I kind of wasn’t talking to anyone, I was just sitting at home by myself for a long time. I mean it was kind of good practice for being a new mum, because you know, there is a lot of sitting at home with someone who can’t really talk. But I think yeah, the connection was probably something that I missed” [Mia, Interview]*.

Mia was also concerned about the inconsistent hospital guidance on the presence and number of support people she could have during labour. As Mia had shared care with her GP, she was subject to a fragmented **model of care** when she was in the hospital system and seeing different midwives at hospital appointments. Therefore, Mia’s biggest concern in her recordings was who she could have supporting her during labour and birth. Mia wanted her husband and mother (a midwife) to support her and wrote a letter of application to the birth unit Midwifery Unit Manager to be allowed a second support for labour.

*“…and then I say* [reads out letter] *“As you can see the first 2 months of dealing with the hospital gave me no faith that I would have been taken care of properly. Over the course of this pregnancy, I have seen five different care providers for antenatal appointments … none of which will be able to continue my care through my birth. I understand the crucial role a trusted care provider and continuity of care can play in labour and birth, even outlined on the hospital website, and I feel I have been denied this through no fault of my own. Based on this information I would like consideration for a second birth partner present, my second birth partner has a background in maternal care and has been supporting me through my pregnancy. As I am currently 38 weeks pregnant please let me know if you require more information to make a decision.” So I sent that in at 38 weeks, and at 40 weeks I got an email saying approved, please print his email out and bring it in for your second birth partner to come along” [Mia, Interview]*.

The letter to the midwifery manager resulted in Mia’s mother being permitted to support her at the birth, alongside her husband. After the birth Mia had limited visitors.

*“only my husband. No one could visit me I couldn’t even get any deliveries of, flowers or balloons or anything like that… and once mum left the hospital, she couldn’t come back in” (Mia interview)*.

Postnatally, Mia **felt anxious** and unsupported as her home **environment** was impacted by lockdowns. Mia thought she would have appreciated having more visitors, as this would allow her to be more enthusiastic about her new baby. Although Mia did connect socially online with other pregnant women and eventually new mothers, these connections were superficial and not long lasting, “*I think if you haven’t met people before, I think a lot of the women found it hard to just go along and meet random people that, have sent a couple of text messages” [Mia, Interview]*.

Overall, Mia **felt anxious** and **unsupported** during her pregnancy and postnatal period, serving to shape Mia’s experience as a new mother as one of a lonely struggle. In a final poignant comment, Mia was asked if she could see any positives in the circumstances of being pregnant during the COVID-19 pandemic, *“I would have liked to not be so alone in this. Yeah, I don’t really see any other positives [laugh] sorry” [Mia, Interview]*.

## Discussion

To the best of our knowledge, this study is the first to examine the real-time experiences of women traversing their maternity care during the COVID-19 pandemic in Australia. In this study, we found women either became lost in a fragmented maternity care system or chose midwifery continuity of care (CoC) models, including homebirth options. In this study the model of care and environment impacted on women’s feelings of anxiety in the perinatal period during the COVID-19 pandemic.

Around one in five women experience perinatal anxiety and depression [26]. Perinatal mental health concerns have far-reaching implications for the mother, baby and society as a whole. An international systematic literature review on perinatal mental health during COVID-19 found eleven studies reported elevated levels of mental health symptoms such as depression and anxiety [27]. Perinatal mental health issues contribute to a range of psychosocial effects including substance abuse, physical health complications, domestic abuse and suicide [28], and the child more likely to go on to develop poor mental health in adolescence [29–32].

These findings are consistent with Stulz et al. (2022), where midwives reported higher anxiety in women and their families during the pandemic, with isolation and unpreparedness key factors contributing to heightened anxiety [33]. Quantitative analysis of women at two months postpartum as part of the mixed methods BITTOC study found women with low/neutral resilience or low/moderate tolerance of uncertainty, a negative cognitive appraisal and greater objective hardship was associated with higher anxiety in the postpartum period [34]. Maternal cognitive appraisal was seen as negative if the woman responded to the question on the consequences of COVID-19 on themselves and their household with a negative instead of positive response and has been associated with postnatal depression [35] and childhood outcomes [36–38]. In additional to these psychological protective factors, this study identified protective factors in the woman’s environment.

### Protective factors of continuity of care

A further finding was that model of care along with environment (hospital, home, society) impacted on anxiety levels. There is a growing body of evidence on the benefits of midwifery CoC models including higher maternal satisfaction [39], with these models characterised by individualised care and trust, allowing women to take the lead in decision-making [40, 41]. Taking a feminist research lens it is important to note that midwifery CoC models challenge the traditional patriarchal, hierarchical models of care by placing women at the centre [42] and working within a partnership paradigm [43, 44].

Women receiving full CoC during the pandemic experienced fewer changes in care compared with standard fragmented models and were more likely to view these as positive [35, 45]. Homebirth during the pandemic has also been found to influence women’s experiences positively [45]. In this study, women either sought out midwifery CoC or experienced a fragmented maternity care system in chaos due to the pandemic. Internationally, this chaos has led to limited support people and doulas in labour, restricted access to waterbirth, closures of maternity services and increased interest in out of hospital birthing [33, 46, 47]. Our findings suggest that midwifery CoC had psychosocial benefits during a pandemic. Midwifery CoC has been found to mitigate the impact of stress on women during natural disasters, as was found in the Queensland flood study [48]. Midwifery CoC should be more readily available for all women including during times of stress and change, such as the pandemic, and the models should be valued as essential services rather than vulnerable to staff relocation and closures.

### Impact of social support on anxiety

Social support was an important factor in how these women experienced the pandemic. Research from before the pandemic found low levels of social support from family, friends and partners and work colleagues have significant effects on increasing anxiety and depression [49, 50]. An analysis of depression, social support and COVID-19 experiences from the BITTOC survey in 2020 found that lower social support from friends and family, and greater family stress or discord were associated with elevated self-reported depressive symptoms in perinatal women [11]. The women in this study reported higher anxiety levels when they had perceived lower levels of social support; however, for some women such as Olivia, being at home in a family bubble with limited visits from friends and family reduced her anxiety around bringing infection into the home environment with a new baby.

### Strengths and limitations

A strength of this study was the utilisation of the Voqual app which allowed the immediate and remote collection of data from participants located across Australia in a time when many research projects were cancelled due to social distancing requirements. A benefit of using the Voqual app was collecting real time data that captured the evolving story. In keeping with the feminist framework women were able to choose when and where they made recordings, and interviews were undertaken online at a time most suited to them. Real-time data collection also prevents the issue of recall bias when relying on postnatal interviews alone [9], which is pertinent considering chronic stress has been shown to impair memory retrieval, owing to high cortisol levels [10]. Women were not given guidance on the topics to record which may have resulted in women recording negative rather than positive experiences.

Women who participated were from higher socio-economic groups, with no First Nations or culturally and linguistically diverse women represented. Additionally, as narrative analysis was used to analyse in depth the stories of the women, there are other stories from participants that, while analysed, could not be captured in this article due to word length.

## Conclusion

The COVID-19 pandemic presented challenges internationally. This study explored the experiences of women giving birth in Australia during the pandemic. Both the maternity model of care and environment had an influence on levels of anxiety in the perinatal period. Our findings highlight the benefits of a supportive continuity of care model for women during times of increased stress and change, such as a pandemic.

## Data Availability

The data that support the findings of this study are available on request from the corresponding author HK. The data are not publicly available due to them containing information that could compromise research participant privacy/ consent.

## References

[1] Fisher JR, Tran TD, Hammarberg K, Sastry J, Nguyen H, Rowe H (2020). Mental health of people in Australia in the first month of COVID-19 restrictions: a national survey. Med J Aust.

[2] Lebel C, MacKinnon A, Bagshawe M, Tomfohr-Madsen L, Giesbrecht G (2020). Elevated depression and anxiety symptoms among pregnant individuals during the COVID-19 pandemic. J Affect Disord.

[3] Chivers BR, Garad RM, Boyle JA, Skouteris H, Teede HJ, Harrison CL (2020). Perinatal distress during COVID-19: thematic analysis of an online parenting forum. J Med Internet Res.

[4] Cooper M, King R. Women’s experiences of maternity care during the height of the COVID-19 pandemic in Australia. Australian College of Midwives; 2020.

[5] Wilson AN, Sweet L, Vasilevski V, Hauck Y, Wynter K, Kuliukas L et al. Australian women’s experiences of receiving maternity care during the COVID-19 pandemic: a cross‐sectional national survey. Birth. 2021. 10.1111/birt.12569 PMC8444895 34180087

[6] Bradfield Z, Hauck Y, Homer CSE, Sweet L, Wilson AN, Szabo RA (2022). Midwives’ experiences of providing maternity care during the COVID-19 pandemic in Australia. Women Birth.

[7] McLeish J, Harrison S, Quigley M, Alderdice F (2022). Learning from a crisis: a qualitative study of the impact on mothers’ emotional wellbeing of changes to maternity care during the COVID-19 pandemic in England, using the National Maternity Survey 2020. BMC Pregnancy Childbirth.

[8] Keedle SV, Burns E, Dahlen H (2018). The design, Development, and evaluation of a qualitative data Collection application for pregnant women. J Nurs Scholarsh.

[9] Khare SR, Vedel I (2019). Recall bias and reduction measures: an example in primary health care service utilization. Fam Pract.

[10] Hidalgo V, Pulopulos MM, Salvador A (2019). Acute psychosocial stress effects on memory performance: relevance of age and sex. Neurobiol Learn Mem.

[11] Lequertier B, McLean MA, Kildea S, King S, Keedle H, Gao Y et al. Perinatal depression in australian women during the COVID-19 pandemic: the birth in the Time of COVID-19 (BITTOC) study. Int J Environ Res Public Health. 2022;19(9). 10.3390/ijerph19095062 PMC9103175 35564456

[12] Curry L, Nunez-Smith M (2015). Mixed methods in Health Sciences Research: a practical primer.

[13] Plano Clark VL, Ivankova NV (2016). Mixed methods research: a guide to the field.

[14] Hesse-Biber SN, ebrary MyiLibrary (2010). Mixed methods research: merging theory with practice.

[15] Ramazanoğlu C, Holland J. Feminist Methodology2002.

[16] Burns E, Fenwick J, Schmied V, Sheehan A (2012). Reflexivity in midwifery research: the insider/outsider debate. Midwifery.

[17] Whitson R (2016). Painting pictures of ourselves: researcher subjectivity in the practice of Feminist Reflexivity. Prof Geogr.

[18] Hoffmann T, Bennett S, Del Mar CB. Evidence-based practice across the health professions-E-pub. Elsevier Health Sciences; 2017.

[19] Joyce M (2015). Using narrative in nursing research. Nurs Standard (Royal Coll Nurs (Great Britain): 1987).

[20] McEnery A (2014). Female doctoral students’ experiences of the Impostor Phenomenon: an exploration of narratives.

[21] Riessman CK. Narrative methods for the human sciences. Sage; 2008.

[22] Keedle H (2020). The experiences of women planning a vaginal birth after caesarean (VBAC) in Australia.

[23] McKelvey M. Narrative analysis: a qualitative method for positive social change. In: Chesnay M, editor. Nursing research using data analysis: qualitative designs and methods in nursing. Springer Publishing Company; 2014.

[24] Keedle H, Schmied V, Burns E, Dahlen HG (2019). A narrative analysis of women’s experiences of planning a vaginal birth after caesarean (VBAC) in Australia using critical feminist theory. BMC Pregnancy Childbirth.

[25] Venla O (2013). Narrative analysis as a feminist method: the case of genetic ancestry tests. Eur J Women’s Stud.

[26] Centre of Perinatal Excellence. Mental health care in the perinatal period Australian clinical practice guideline. 2017.

[27] Iyengar U, Jaiprakash B, Haitsuka H, Kim S (2021). One year into the pandemic: a systematic review of Perinatal Mental Health Outcomes during COVID-19. Front Psychiatry.

[28] Howard LM, Khalifeh H (2020). Perinatal mental health: a review of progress and challenges. World Psychiatry.

[29] Gentile S (2017). Untreated depression during pregnancy: short- and long-term effects in offspring. A systematic review. Neuroscience.

[30] Davies SM, Silverio SA, Christiansen P, Fallon V (2021). Maternal-infant bonding and perceptions of infant temperament: the mediating role of maternal mental health. J Affect Disord.

[31] Ding XX, Wu YL, Xu SJ, Zhu RP, Jia XM, Zhang SF (2014). Maternal anxiety during pregnancy and adverse birth outcomes: a systematic review and meta-analysis of prospective cohort studies. J Affect Disord.

[32] Lewis AJ, Austin E, Knapp R, Vaiano T, Galbally M (2015). Perinatal maternal mental health, fetal programming and child development.

[33] Stulz VM, Bradfield Z, Cummins A, Catling C, Sweet L, McInnes R (2022). Midwives providing woman-centred care during the COVID-19 pandemic in Australia: a national qualitative study. Women Birth.

[34] Di Paolo AL, King S, McLean MA, Lequertier B, Elgbeili G, Kildea S (2022). Prenatal stress from the COVID-19 pandemic predicts maternal postpartum anxiety as moderated by psychological factors: the australian BITTOC Study. J Affect Disord.

[35] Cummins A, Baird K, Melov SJ, Melhem L, Hilsabeck C, Hook M et al. Does midwifery continuity of care make a difference to women with perinatal mental health conditions: a cohort study, from Australia. Women Birth. 2022. 10.1016/j.wombi.2022.08.002 35941058

[36] Dancause KN, Laplante DP, Fraser S, Brunet A, Ciampi A, Schmitz N (2012). Prenatal exposure to a natural disaster increases risk for obesity in 5(1/2)-year-old children. Pediatr Res.

[37] Veru F, Dancause K, Laplante DP, King S, Luheshi G (2015). Prenatal maternal stress predicts reductions in CD4 + lymphocytes, increases in innate-derived cytokines, and a Th2 shift in adolescents: Project Ice Storm. Physiol Behav.

[38] Dancause KN, Veru F, Andersen RE, Laplante DP, King S (2013). Prenatal stress due to a natural disaster predicts insulin secretion in adolescence. Early Hum Dev.

[39] Sandall J, Soltani H, Gates S, Shennan A, Devane D (2016). Midwife-led continuity models versus other models of care for childbearing women. Cochrane Database Syst Rev.

[40] Perriman N, Davis DL, Ferguson S (2018). What women value in the midwifery continuity of care model: a systematic review with meta-synthesis. Midwifery.

[41] Keedle H, Peters L, Schmied V, Burns E, Keedle W, Dahlen HG (2020). Women’s experiences of planning a vaginal birth after caesarean in different models of maternity care in Australia. BMC Pregnancy Childbirth.

[42] Hawke M (2021). Subversive acts and everyday midwifery: feminism in content and context. Women Birth.

[43] Boyle S, Thomas H, Brooks F (2016). Women’s views on partnership working with midwives during pregnancy and childbirth. Midwifery.

[44] Walsh D (1999). An ethnographic study of women’s experience of partnership caseload midwifery practice: the professional as a friend. Midwifery.

[45] Kluwgant D, Homer C, Dahlen H (2022). Never let a good crisis go to waste: positives from disrupted maternity care in Australia during COVID-19. Midwifery.

[46] Dahlen HG, Kumar-Hazard B, Chiarella M, How (2020). COVID-19 highlights an ongoing pandemic of neglect and oppression when it comes to Women’s Reproductive Rights. J Law Med.

[47] Davis-Floyd R, Gutschow K, Schwartz DA, Pregnancy (2020). Birth and the COVID-19 pandemic in the United States. Med Anthropol.

[48] Kildea S, Simcock G, Liu A, Elgbeili G, Laplante DP, Kahler A (2018). Continuity of midwifery carer moderates the effects of prenatal maternal stress on postnatal maternal wellbeing: the Queensland flood study. Arch Women Ment Health.

[49] Sufredini F, Catling C, Zugai J, Chang S. The effects of social support on depression and anxiety in the perinatal period: a mixed-methods systematic review. J Affect Disord. 2022. 10.1016/j.jad.2022.09.005 36108877

[50] Milgrom J, Hirshler Y, Reece J, Holt C, Gemmill AW. Social Support-A Protective factor for depressed Perinatal Women? Int J Environ Res Public Health. 2019;16(8). 10.3390/ijerph16081426 PMC6518117 31010090

